# Lytic Polysaccharide Monooxygenases as Chitin-Specific Virulence Factors in Crayfish Plague

**DOI:** 10.3390/biom11081180

**Published:** 2021-08-09

**Authors:** Federico Sabbadin, Bernard Henrissat, Neil C. Bruce, Simon J. McQueen-Mason

**Affiliations:** 1 Centre for Novel Agricultural Products, Department of Biology, University of York, York YO10 5DD, UK; neil.bruce@york.ac.uk; 2 DTU Bioengineering, Technical University of Denmark, Søltofts Plads, 2800 Kongens Lyngby, Denmark; bernard.henrissat@gmail.com; 3 Department of Biological Sciences, King Abdulaziz University, Jeddah 21589, Saudi Arabia

**Keywords:** crayfish plague, oomycete, lytic polysaccharide monooxygenase, chitin, pathogen, infection, virulence

## Abstract

The oomycete pathogen *Aphanomyces astaci*, also known as “crayfish plague”, is an obligate fungal-like parasite of freshwater crustaceans and is considered responsible for the ongoing decline of native European crayfish populations. *A. astaci* is thought to secrete a wide array of effectors and enzymes that facilitate infection, however their molecular mechanisms have been poorly characterized. Here, we report the identification of AA15 lytic polysaccharide monooxygenases (LPMOs) as a new group of secreted virulence factors in *A. astaci*. We show that this enzyme family has greatly expanded in *A. astaci* compared to all other oomycetes, and that it may facilitate infection through oxidative degradation of crystalline chitin, the most abundant polysaccharide found in the crustacean exoskeleton. These findings reveal new roles for LPMOs in animal–pathogen interactions, and could help inform future strategies for the protection of farmed and endangered species.

## 1. Introduction

Oomycetes are a wide group of filamentous microorganisms phylogenetically related to brown algae, and include some of the most damaging pathogens in modern agriculture and aquaculture [1]. The oomycete order *Saprolegniales* has attracted attention due to its ubiquitous occurrence and destructive effects on fish farms as we well as natural salmonid and crayfish populations [2]. This group of oomycetes includes *Aphnomyces astaci*, an obligate parasite of freshwater decapod crustaceans that was accidentally introduced, through commercial trade, from North America into Europe in the mid nineteenth century [3]. Since then, *A. astaci* has rapidly spread throughout Europe, decimating native crayfish populations, which show no resistance to the infection as opposed to resistant signal crayfish found in America. *A. astaci* is often referred to as “crayfish plague” due to its devastating impact on noble crayfish (*Astacus astacus*), now listed as a vulnerable species on the IUCN Red List [4]. Current conservation efforts through monitoring and reintroduction in restored habitats have had limited success, and noble crayfish populations are steadily declining.

*A. astaci* spreads by releasing flagellate zoospores [5] that swim in aquatic environments and follow chemotaxis trails towards potential hosts, predominantly crustaceans. Upon contact with the host, the zoospores encyst and form a peg that penetrates the host’s cuticle through a combination of mechanical force and enzymatic degradation. The crustacean exoskeleton represents a major barrier to the pathogen, and is the site where the molecular struggle between pathogen and host commences [6]. The exoskeleton is composed of a calcified multi-layered extracellular matrix, which is the cuticle, consisting of chitin, proteins, minerals and lipids [7]. Chitin is the primary structural polysaccharide in the crustacean exoskeleton, and is composed of long chains of β-1,4 linked N-acetylglucosamine units stabilized by intra- and inter-chain hydrogen bonds that confer strength and rigidity to the crystalline polymer. In order to penetrate the host’s cuticle and invade the underlying tissue, growing hyphae of *A. astaci* are thought to secrete several types of lytic enzymes, including proteases, lipases and carbohydrate active enzymes (CAZymes) [6]. While most research on *Saprolegniales* has focused on secreted proteases and intracellular effectors (e.g., Crinkler factors [6]), the role of CAZymes in infection has been overlooked.

Little work has been carried out on the biochemical characterization of enzymes secreted by *A. astaci*. Northern blot and RT-PCR analysis of one *A. astaci* gene coding for a glycoside hydrolase family 18 (GH18, chitinase) revealed that expression was very low at zoospore stage but increased at late germination, reaching strong constitutive expression in growing mycelium (vegetative growth). The authors saw no further induction by addition of chitin in the medium, and concluded that the observed expression pattern of the chitinase is likely an adaptation to the exclusively parasitic lifestyle of this species [8]. Increased chitinase activity detected in the growth medium during mycelial growth further supported the notion that chitinases are secreted by the hyphae to weaken the host’s exoskeleton and facilitate penetration [8]. Bioinformatic analysis has indeed evidenced the abundance of putative chitinases in the genome of *A. astaci* compared to the plant pathogen *Aphanomyces euteiches*, suggesting a role for these enzymes in crayfish plague and reflecting its strict host specialization [6,9,10].

The genomes of oomycetes harbor numerous genes encoding CAZymes, including auxiliary activity (AA) proteins that act in conjunction with canonical glycoside hydrolases (GHs). In the CAZy database, eight AA families are represented by mononuclear copper enzymes called lytic polysaccharide monooxygenases (LPMOs), which catalyze the oxidative cleavage of glycosidic bonds in polysaccharides (cellulose, chitin, starch, xylan, xyloglucan, glucomannan, homogalacturonan) [11,12,13,14]. LPMOs greatly synergize the activity of glycoside hydrolases and have been included into commercial enzymatic cocktails to enhance plant biomass saccharification towards the production of second-generation bioethanol. In order to carry out this task, LPMOs require external electron donors (e.g., small phenolic compounds or ascorbic acid [15]) or redox protein partners (e.g., cellobiose dehydrogenase in fungi [16]). Although theoretical calculations show that molecular oxygen plus one electron reduction lead to the energetically favorable formation of a copper peroxide intermediate and insertion of an oxygen atom into the glycoside substrate, recent work has also shown that LPMOs may alternatively use hydrogen peroxide both as a co-substrate and source of electrons [17,18].

Current hypotheses on the mechanisms of pathogenesis in *A. astaci* mostly rely on genomic surveys and predicted functional annotation of putative proteins, leaving a gap in our understanding of the biochemical properties and physiological roles of CAZymes during infection. Here, we show that *A. astaci* harbors an expanded family of LMPOs and provide evidence of its role in breaching the tough exoskeleton of crayfish. Through phylogeny and biochemical studies, we show that these LPMOs have been co-opted by *A. astaci* as secreted virulence factors to cleave crystalline chitin through oxidative attack, thereby facilitating host tissue invasion. These findings expand our knowledge of the biological roles of LPMOs in Eukaryotes and their involvement in animal pathogenesis, and could help develop new approaches towards the protection of species threatened by oomycetes.

## 2. Materials and Methods

### 2.1. Protein Sequence Annotation, Alignment and Phylogeny

The proteomes of *Aphanomyces astaci* (accession PRJNA264335), *Achlya hypogyna* (accession PRJNA169234), *Aphanomyces invadans* (accession PRJNA258292), *Saprolegnia diclina* (accession PRJNA255245), *Saprolegnia parasitica* (accession PRJNA280969) and *Thraustotheca clavata* (accession PRJNA169235) were annotated using the online tool dbCAN2 [19]. AA15 sequences were retrieved, and the signal peptides and C-terminal extensions were removed to avoid interference from the presence or absence of additional modules. The resulting amino acid sequences corresponding to the catalytic domain were aligned using T-Coffee [20], operating with default parameters. A phylogenetic tree was built with MegaX [21] using the maximum likelihood method. The resulting tree was visualized using Dendroscope [22] and edited with the graphic tool CorelDraw Graphics Suite 2020. C-terminal extensions of AA15 sequences from *A. astaci*, *A. hypogyna*, *S. diclina* and *S. parasitica*, identified as members of the X473 and X474 families based on Hidden Markov Models (HMMs), were aligned using T-Coffee [20] and visualized using Jalview [23].

### 2.2. RNAseq Analysis of A. astaci at Different Stages of the Life Cycle

Raw transcriptomic Illumina HiSeq 2000 data from *A. astaci* sporulating mycelia, growing mycelia and zoospores were retrieved from accessions SRX236908, SRX236909 and SRX236910. Raw reads were mapped onto the published transcriptome of *A. astaci* (accession PRJNA264335) to determine normalized expression values (TPM = transcripts per kilobase million) using Salmon (part of the Galaxy toolshed) [24,25].

### 2.3. Homology Modelling

The Swiss-Model [26] server (https://swissmodel.expasy.org/, accessed on 10 March 2021) was used to build a homology model of *Aa*AA15a using the structure of *Td*AA15A (PDB code: 5MSZ) as a template. The analysis gave a GQME score of 0.58 and a QMEAN Z-score of −2.41, which are reasonable for a useful model. Structure figures were developed using CCP4mg [27].

### 2.4. Cloning and Heterologous Expression of AaAA15A

The codon optimized sequence for the LPMO catalytic domain of *Aa*AA15A was amplified with oligonucleotide primers using Phusion DNA Polymerase (Thermo Fisher Scientific, Waltham, MA, USA). Following the addition of a C-terminal Strep-tag^®^ II (WSHPQFEK), the PCR product was cloned into pET22b in frame with the pelB leader sequence (required to direct the protein to the *E. coli* periplasmic space) using the InFusion^®^ HD Cloning Kit (Takara Bio, Kusatsu, Shiga, Japan). The expression plasmid carrying the cloned sequence was transformed into *Escherichia coli* Rosetta 2 (DE3) pLysS (Merck, Darmstadt, Germany) via heat shock. One colony was used to inoculate 10 mL of LB medium with antibiotics (100 μg mL^−1^ ampicillin and 34 μg mL^−1^ chloramphenicol) and was grown overnight at 30 °C at 180 rpm. This starter culture was then used to inoculate 1 L of M9 minimal salts supplemented with 1% (*w*/*v*) glucose as carbon source, plus antibiotics (100 μg mL^−1^ ampicillin and 34 μg mL^−1^ chloramphenicol). The cell culture was grown at 37 °C in a shaker at 210 rpm, induced with 1 mM IPTG when the OD600 reached 0.5, and left shaking at 210 rpm overnight at 16 °C. The cells were harvested through centrifugation, re-suspended in 50 mL ice cold 50 mM Tris HCl pH 8 with 20% (*w*/*v*) sucrose, and left in ice for 30 min. The suspension was centrifuged, the supernatant was discarded and the cell pellet was re-suspended in 40 mL ice cold 5 mM MgSO_4_ supplemented with 100 µM AEBSF protease inhibitor and incubated in ice for 30 min. After another round of centrifugation, the supernatant was collected, filtered with 0.22 µm filters, and the pH adjusted to 7.6 with 50 mM Na phosphate buffer (binding buffer). The subsequent purification steps were carried out with an AKTA™ FPLC system (Cytiva). The clarified periplasmic extract was injected into a 5 mL StrepTrap HP column (Cytiva), followed by washing with binding buffer, and the protein was eluted with 2.5 mM desthiobiotin in binding buffer. Protein concentration was measured with a NanoDrop spectrophotometer based on the absorbance at 280 nm, the molecular weight and the extinction coefficient of the mature protein. Following the addition of fivefold molar excess copper (CuSO_4_), the protein was passed in a HiLoad™ 16/60 Superdex 75 gel filtration column (Cytiva) equilibrated with 20 mM Tris-HCl buffer pH 7, in order to remove unbound excess copper and desthiobiotin. The protein was then concentrated through centrifugation using Microsep™ Advance Centrifugal Devices (Pall Corporation) and quantified again with a NanoDrop spectrophotometer.

### 2.5. Thermal Shift Assay (Thermofluor)

The purified, copper-loaded *Aa*AA15a was analyzed through thermal shift (Thermofluor) assay, using SYPRO^®^ Orange Protein Gel Stain ( Thermo Fisher Scientific, Waltham, MA, USA). The intensity of the fluorescence was measured with an Mx3005P qPCR System (Agilent Technologies, Santa Clara, CA, USA) across a temperature gradient from 25 to 95 °C, and converted into a melting curve (fluorescence changes against temperature) to determine the melting temperature (*T_m_*) value.

### 2.6. In Vitro Activity Assays

Reactions were carried out by mixing 4 mg mL^−1^ substrate with purified copper-loaded *Aa*AA15A (2 μM), 4 mM electron donor (gallic acid), in 50 mM ammonium acetate buffer with a total volume of 100 μL in 2 mL plastic reaction tubes. Reactions were incubated for 24 h at 28 °C shaking at 1000 rpm, then tubes were centrifuged at 20,817× *g* and the supernatant was collected for analysis through mass spectrometry (see paragraph “Product analysis by mass spectrometry”).

### 2.7. Product Analysis by Mass Spectrometry

Reactions were centrifuged at 20,817× *g*, one microliter of supernatant was mixed with an equal volume of matrix solution (20 mg mL^−1^ 2,5-dihydroxybenzoic acid (DHB) in 50% acetonitrile plus 0.1% TFA) and spotted on a SCOUT-MTP 384 target plate (Bruker, Billerica, MA, USA). The spotted samples were air dried and then analyzed by positive-mode MALDI-TOF MS using an Ultraflex III matrix-assisted laser desorption ionization-time of flight/time of flight (MALDI/TOF-TOF) instrument (Bruker).

## 3. Results

### 3.1. Experimental Results

#### 3.1.1. Gene Copy Numbers of LPMO Families in Oomycetes Correlate with Host Preference and Life Style

Eight LPMO families spanning across fungi, viruses, bacteria, invertebrates and oomycetes have been identified to date, and have mostly been studied in the context of plant biomass breakdown. Oomycetes carry three LPMO families (AA15, AA16 and AA17), but only members of the AA17 family have been characterized so far, showing activity on pectin from plant cell walls [28].

**Figure 1:**
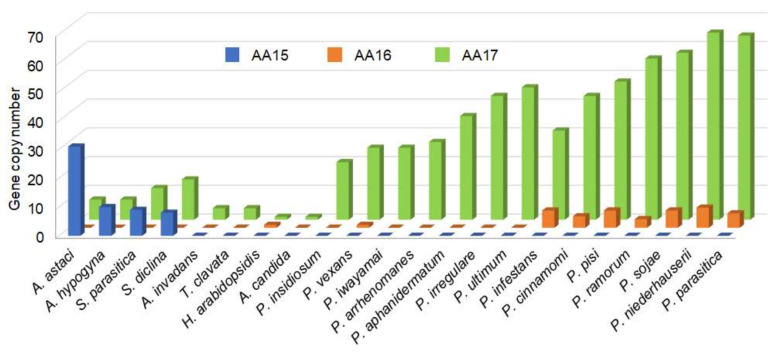
Abundance of LPMO coding genes in oomycete species. AA: Auxiliary Activity.

In order to elucidate the distribution and potential roles of LPMOs and other CAZymes in *Saprolegniales*, we carried out predicted functional annotation of oomycete proteomes using dbCAN2, and noticed that oomycetes with different host preference harbor distinct arrays of LPMOs. *Saprolegniales* (mostly animal pathogens) carry families AA15 [14] and AA17 [28], while *Peronosporales* and *Pythiaceae* (mostly plant pathogens, *Phytophthora* and *Pythium* species) carry families AA16 [29] and AA17 [28]. We also noticed that gene copy numbers correlate with oomycete lifestyles. In our previous work, we showed that AA17 LPMOs have greatly expanded in necrotrophic plant pathogenic oomycetes compared to biotrophic ones (averaging 46 copies in *Phytophthora* spp.), and are secreted as virulence factors for plant cell wall disruption [28]. AA17 genes are much less abundant (eight copies on average) in *Saprolegniales* infecting animals (Figure 1), where they are typically fused to cellulose-binding domains [28] and likely carry out remodelling of endogenous cellulose in the oomycete cell wall.

**Figure 2:**
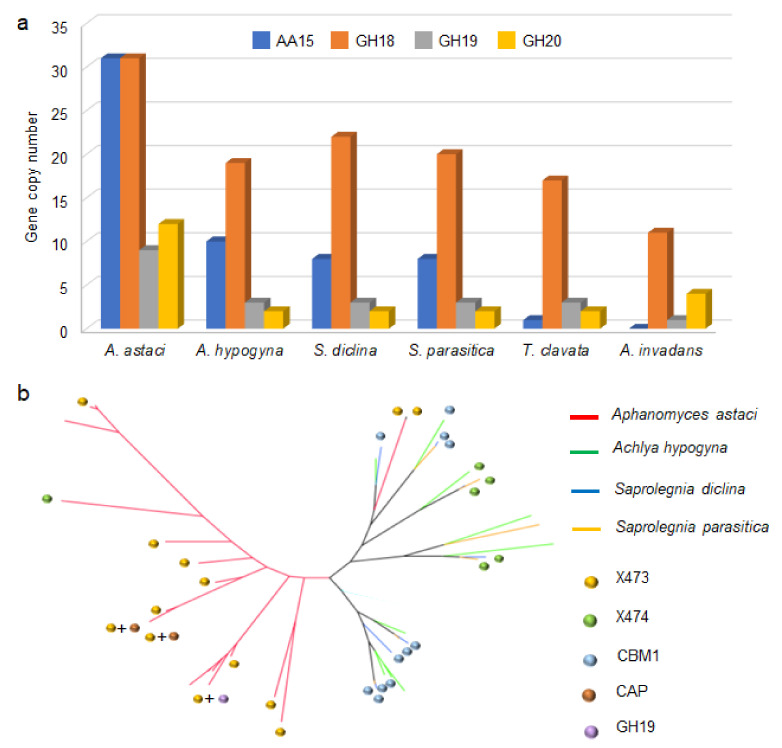
Abundance of chitin-active CAZy families in oomycetes and phylogeny of AA15 LPMOs in zoonotic species. (**a**) Histogram illustrating the number of genes coding for AA15 (Auxiliary Activity Family 15), GH18 (glycoside hydrolase family 18, chitinase), GH19 (glycoside hydrolase family 19, chitinase/lysozyme) and GH20 (glycoside hydrolase family 20, β-N-acetylglucosaminidase) in representative *Saprolegniales*. (**b**) Maximum likelihood phylogenetic tree of AA15 LPMOs (catalytic domain only) from four major zoonotic *Saprolegniales* species (*A. astaci*, *A. hypogyna*, *S. diclina* and *S. parasitica*). Annotations indicate additional domains C-terminally fused to the AA15 domain. The most common extension of AA15s in *A. hypogyna*, *S. diclina* and *S. parasitica* is represented by CBM1, while in *A. astaci* it is X473. CBM1: carbohydrate binding module 1 (cellulose specific). CAP: cysteine-rich secretory proteins, antigen 5 and pathogenesis-related 1 proteins [34].

Inspection of predicted oomycete proteomes for the presence of genes encoding hydrolytic chitinases revealed a significant expansion of putative endo-chitinases (GH18, GH19) [30,31] and exo-chitinases (GH20) [32] in A. astaci compared to all other oomycetes (Figure 2a). A similar pattern was observed for the AA15 family in Saprolegniales. These LPMOs are absent in *Peronosporales* and *Pythiaceae* (Figure 1), and their gene copy numbers in *Saprolegniales* appear to be linked to host preference (Figure 2a). *Aphanomyces invadans* (obligate pathogen of bony fish and the causative agent of Epizootic Ulcerative Syndrome) has no AA15 genes. Only one copy is found in *Thraustotheca clavata* (free living non-pathogenic saprobe). There are eight copies in *Saprolegnia parasitica* and *Saprolegnia diclina* (which can infect fish, fish eggs, amphibians and crustaceans), ten copies in *Achlya hypogyna* (facultative crustacean decapod parasite) and thirty-one copies in *A. astaci* (preferential parasite of crustacean decapods). AA15 gene copy number is, therefore, much higher in necrotrophic species that infect aquatic animals, particularly crustaceans. This trend suggests a specialized role for these LPMOs in attacking a protective structural component abundant in crustaceans yet absent or scarce in plants and bony fish, which we speculated could be chitin. Supporting this hypothesis, in the genome of *A. astaci*, we identified a genomic island composed of nine identical AA15 genes (spanning from H257_10979 to H257_10987), similar to bacterial high-density pathogenicity islands (PAIs) typically involved in infection [33]. Phylogenetic analysis also shows that *A. astaci* AA15 sequences cluster separately from those found in other *Saprolegniales* (Figure 2b).

#### 3.1.2. Functional Domain Annotation Supports a Specialized Role for *A. astaci* AA15s in Chitin Degradation

**Figure 3:**
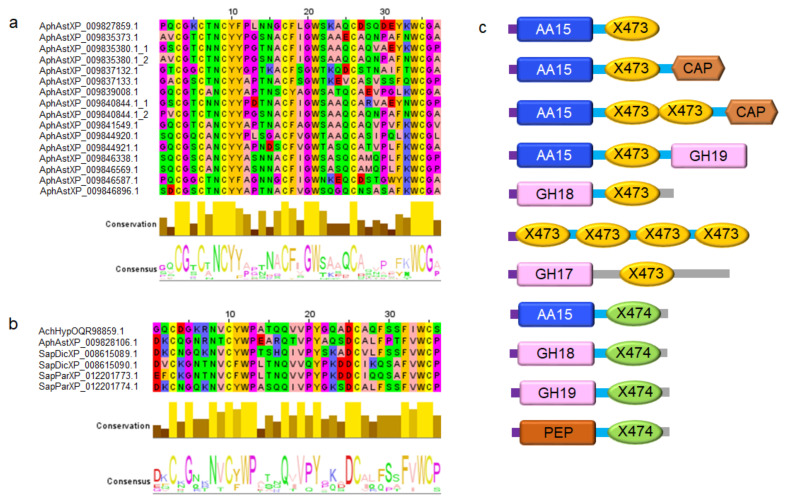
Multiple sequence alignment and architecture of X473 and X474 domains. (**a**) Alignment of X473 sequences found to be fused to AA15 LPMOs in *A. astaci*. (**b**) Alignment of X474 sequences found to be fused to AA15 LPMOs from *A. astaci*, *A. hypogyna*, *S. diclina* and *S. parasitica*. The alignments show absolute conservation of the cysteine residues (six and four in X473 and X474, respectively) putatively involved in disulfide bond formation. The bottom part of panels a and b also shows the level of conservation and the consensus sequence. Sequences are labelled with the species name (AphAst = *A. astaci*, AchHyp = *A. hypogyna*, SapDic = *S. diclina*, SapPar = *S. parasitica*) followed by the NCBI reference number. (**c**) Domain architecture of proteins carrying the X473 and X474 extensions in *Saprolegniales*. Both X473 and X474 are normally associated with chitinases (GH18, GH19). X474 is occasionally fused to putative M14 metallocarboxypeptidases, C1 cysteine peptidases, and trypsin-like serine proteases (“PEP”). Purple: predicted signal peptide. Light blue: putative linker peptide. Grey: unknown. CBM1: carbohydrate binding module 1 (cellulose-specific). GH17: glycoside hydrolase family 17. GH18: glycoside hydrolase family 18. GH19: glycoside hydrolase family 19. X473 and X474: newly identified domains, putatively involved in binding chitin. CAP = cysteine-rich secretory proteins, antigen 5, and pathogenesis-related 1 proteins superfamily [34].

All full-length AA15 members in *A. astaci* carry a signal peptide for secretion of the mature protein, which features an N-terminal histidine and a second conserved histidine, involved in the formation of a “histidine brace” [12], the hallmark of catalytically active LPMOs. In *A. hypogyna*, *S. diclina* and *S. parasitica,* over half of the LPMO catalytic domains are also fused to a carbohydrate-binding module 1 (CBM1, cellulose-binding) [35]. In *A. astaci*, however, only one out of thirty-one AA15s is fused to a CBM1, whereas sixteen of them are fused to short (<40 residues) C-terminal domains of unknown function. These uncharacterized domains have even numbers of absolutely conserved cysteines (Figure 3a,b), akin to those involved in disulfide bond formation in CBMs and required for proper folding and substrate binding [35]. These domains belong to two new families, henceforth named X473 (featuring six conserved cysteines) and X474 (featuring four conserved cysteines). BlastP searches over NCBI databases show that X473 and X474 domains are only found in *Saprolegniales*, and they are typically fused to canonical hydrolytic chitinases (GH18 and GH19 families [30,31]) (Figure 3c).

#### 3.1.3. AA15 Gene Expression and Homology Modelling

**Figure 4:**
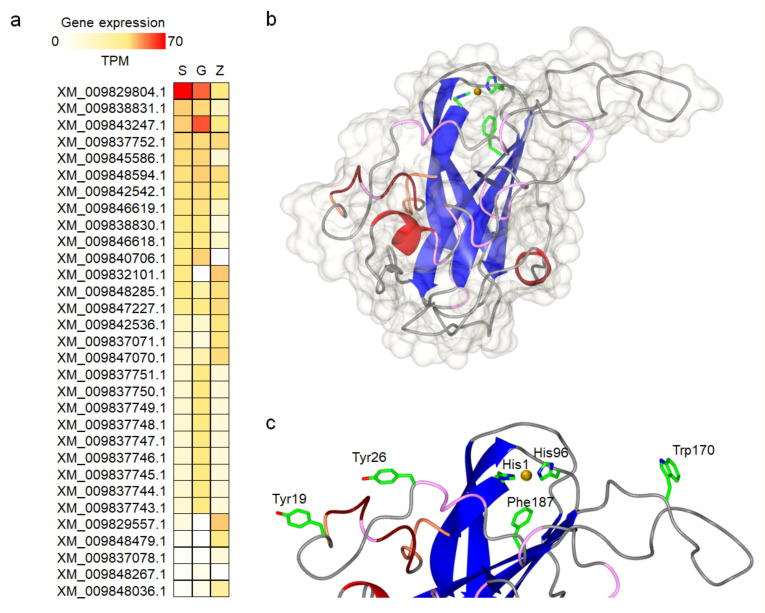
Gene expression of AA15 coding genes in *A. astaci* and structural homology modelling of *Aa*AA15A. (**a**) Relative gene expression levels for all AA15 genes across life stages were calculated using Salmon [25]. The highest expression level is observed for gene XM_009829804.1 (*Aa*AA15A). TPM: transcripts per kilobase million. S: sporulating mycelia. G: growing mycelia. Z: zoospores. Raw data were obtained from accessions SRX236908, SRX236909 and SRX236910. (**b**) Homology model of *Aa*AA15A generated with Swiss-Model using the crystal structure of *Td*AA15A (PDB code: 5MSZ) as template. The model is shown as a cartoon colored by secondary structure with its surrounding surface shown in grey. (**c**) Close up view of the copper-coordinating “histidine brace” and aromatic residues at the substrate-binding surface.

We analyzed publicly available transcriptomic datasets of *A. astaci* (accessions SRX236908, SRX236909 and SRX236910) and identified gene XM_009829804.1 (from now on referred to as *Aa*AA15A) as the most highly expressed AA15-encoding gene in both sporulating and growing mycelia, but low at zoospore stage (Figure 4a). This expression pattern, similar to the one recorded for a GH18 gene in *A. astaci* and linked to the parasitic life-style of this species [8], prompted us the investigate *Aa*AA15A in more detail. The Swiss-Model server [26] was used to build a homology model of *Aa*AA15A based on the X-ray structure of *Td*AA15A (from the insect *Thermobia domestica*) [14], which shares high protein sequence identity (43%). The generated model has the typical central β-sandwich fold of LPMOs, as well as flexible loops and two stabilizing disulfide bonds (Figure 4b). The active site features the ubiquitous LPMO “histidine brace” [12], consisting of His1 and His96 coordinating the copper cofactor, and the axial, non-coordinating residue Phe187, previously suggested to play a role in the degradation of chitin in insect AA15s [14] (Figure 4c). *Aa*AA15A, like *Td*AA15A, has an unusual β-tongue-like protrusion and forms part of the flat surface surrounding the active site. On opposite sides of the histidine brace, and marking the boundaries of the flat surface surrounding the active site, are the aromatic rings of Tyr19, Tyr26 and Trp170, which are likely involved in substrate binding, similarly to other characterized LPMOs [36] (Figure 4c).

#### 3.1.4. Purification and Biochemical Characterization of *Aa*AA15A, a Chitin-Specific LPMO

**Figure 5:**
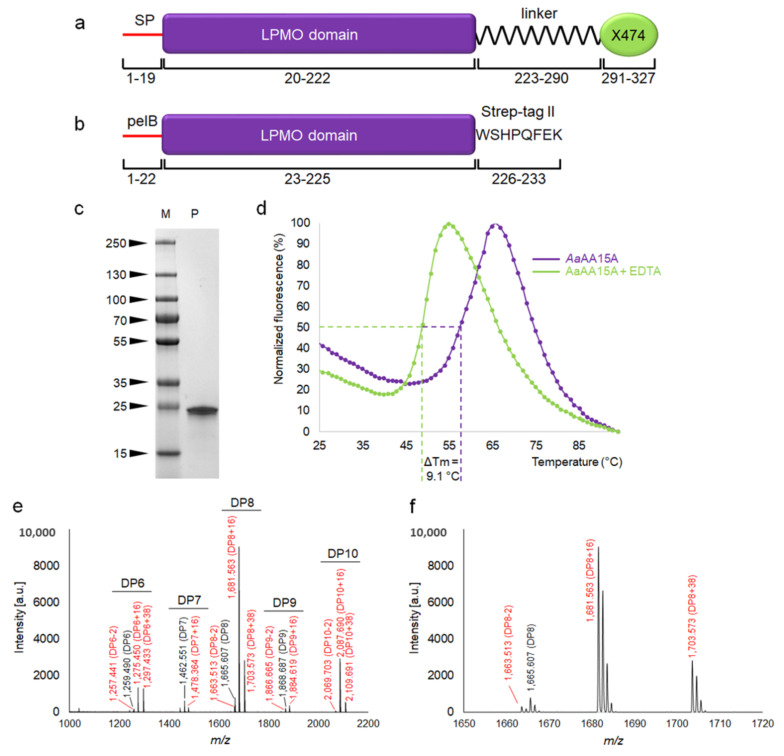
Domain architecture, production, stability and activity of *Aa*AA15A. (**a**) Architecture of the full-length *Aa*AA15A protein, featuring a signal peptide for secretion (SP), an AA15 LPMO domain, a 67-residue long peptide linker and an X474 domain, putatively involved in binding chitin. (**b**) Architecture of the recombinant *Aa*AA15A core used in this study. (**c**) SDS-PAGE of purified *Aa*AA15A (LPMO domain) heterologously produced in *E. coli* (M molecular weight markers in kDa, P purified protein). The expected molecular weight of the mature, strep-tagged protein is 22.9 kDa. The final yield from the purification of *Aa*AA15A was about 1 mg of protein per liter of cell culture. (**d**) Thermal shift analysis of purified *Aa*AA15A LPMO domain, showing the destabilizing effect of copper removal through EDTA treatment, causing a 9.1 °C decrease in the melting temperature. The assay was carried out in a single repeat, with each point calculated as a moving average of three readings to filter out noise from random short-term fluctuations. The ramp rate was 1 °C every 30 s. (**e**) MALDI-TOF MS spectrum of products obtained after incubation of 4 mg mL^−1^ β-chitin with 2 µM *Aa*AA15A and 4 mM gallic acid for 24 h, showing native and oxidized oligosaccharides. The main peaks correspond to mono- or di-sodiated adducts of C1-aldonic acids, imparting +16 or +38 m/z, respectively, relative to the mono-sodiated unoxidized form. Smaller peaks for the mono-sodiated lactone (−2) were also identified. Native and oxidized species are marked in black and red, respectively. (**f**) Expanded mass spectra for DP8 products.

We cloned the codon optimized sequence of the *Aa*AA15A catalytic domain (without the signal peptide and the X474 domain) into a plasmid for periplasmic expression in *E. coli* and purified the C-terminally strep-tagged protein using established methods [14] (Figure 5a–c). Thermal shift (Thermofluor) analysis of purified copper-bound *Aa*AA15A indicates a melting temperature (*T_m_*) of 59.2 °C, which is lowered to 49.3 °C upon addition of 10 mM EDTA (Figure 5d). These results indicate that the apo-enzyme folds correctly in the periplasm of *E. coli* and that copper increases the *T_m_* and protein stability, as observed with other LPMOs [13,14]. Activity assays with purified, copper-loaded *Aa*AA15A were carried out on a panel of polysaccharide substrates (microcrystalline cellulose, phosphoric acid swollen cellulose, shrimp chitin, squid pen chitin) in presence of the electron donor gallic acid. Samples were analyzed by MALDI-TOF MS and peak masses of the reaction products compared to previously published data [11,13,14], revealing a predominant C1-oxidation pattern and generation of C1-aldonic acids from both partially amorphous squid pen chitin (β-chitin, Figure 5e,f) and crystalline shrimp chitin (α-chitin, from shrimp shells, Figure S1) in the presence of the external electron donor. The products released by *Aa*AA15A seem to be predominantly even-numbered oligosaccharides, implying that the enzyme can attack the crystalline structure of chitin [11]. Native and oxidized products were not detected in any of the negative control reactions (substrate alone, substrate plus gallic acid, substrate plus LPMO; data not shown).

## 4. Discussion

Of all zoonotic oomycetes, *A. astaci* is the most specialized in terms of host preference (attacking crustaceans almost exclusively), yet the molecular mechanisms underlying this specificity are poorly understood. In this study, through a combination of protein sequence analysis, phylogeny and biochemical in vitro characterization, we revealed that AA15 LPMOs likely act as virulence factors in *A. astaci*. Our data indicate that these copper-dependent enzymes are part of an expanded arsenal of chitin-degrading enzymes that are secreted upon infection and target the hardest part of the crustacean cuticle, facilitating tissue penetration and infection.

Most research carried out on LPMOs has focused on fungal members and their biotechnological applications in the context of plant biomass valorization and conversion into bioethanol [37]. However, our recent discovery of the first LPMO family (called AA15) in animals, oomycetes and algae revealed the wider taxonomical distribution of these enzymes [14], opening up new lines of investigation. Most invertebrates, algae and oomycetes have between one and five AA15 gene copies; however, some species show remarkable expansion of their AA15 pool, particularly *A. astaci* and the detritivorous insect called *Thermobia domestica*. We previously showed that *T. domestica* expanded its repertoire of AA15s to over twenty isoforms in order to boost its ability to digest plant biomass [14], and our new data indicate that *A. astaci* has also increased its gene copy number as a result of evolutionary adaptation to its parasitic lifestyle. Interestingly, previous work demonstrated that the catalytic domain of fusolin proteins produced by entomopoxyviruses is an AA10 LPMO that boosts the virulence of the pathogen by disrupting of the chitin-rich peritrophic matrix in the insect’s digestive system [38]. It is likely that bacterial, fungal and oomycete pathogens have also adapted LPMOs as weapons to infect arthropods, and our study in *A. astaci* supports this hypothesis.

The crustacean immune response is a complex array of hemocyte-derived (cellular) and plasma-derived (humoral) immune factors. Infection of crayfish by pathogenic oomycetes triggers the hemocyte-mediated prophenoloxidase system and the formation of a melanin sheath around the pathogen, in order to contain it and stop it from spreading into the hemocoel [39]. Melanin spots are typically seen in the infected cuticle of resistant North American crayfish (e.g., *Pacifastacus leniusculus* and *Procambarus clarkii*), but are scarce or absent in vulnerable European species, especially *A. astacus* [39], suggesting a slow hemocyte-mediated response. Could this discrepancy depend on the different abilities of crayfish species to sense cuticle breakdown products and the pathogen’s secreted enzymes, including LPMOs? Plants have evolved several proteinaceous inhibitors (such as polygalacturonase-inhibiting proteins and pectin-methylesterase-inhibiting proteins, PGIP and PMEI) that target and neutralize carbohydrate-active enzymes during fungal infection [40] and attack by herbivorous insect [41]. Further work is needed to determine if inhibitors of carbohydrate-active enzymes have also evolved in complex animals. The identification of LPMO-inhibiting proteins in crayfish could greatly deepen our knowledge of host-pathogen interactions, as well as open up new opportunities towards disease control, protection of endangered species and enhanced food security.

## Data Availability

All data presented are in the main paper and Supplementary Materials. All other data are freely accessible online.

## Supplementary Materials

The following are available online at https://www.mdpi.com/article/10.3390/biom11081180/s1, Figure S1: MALDI-TOF MS of activity assays of *Aa*AA15a with α-chitin.

## References

[1] Derevnina L., Petre B., Kellner R., Dagdas Y.F., Sarowar M.N., Giannakopoulou A., De La Concepcion J.C., Chaparro-Garcia A., Pennington H.G., Van West P. (2016). Emerging oomycete threats to plants and animals. Philos. Trans. R. Soc. B Biol. Sci..

[2] van den Berg A.H., Mclaggan D., Dieguez-Uribeond J., van West P. (2013). The impact of the water moulds Saprolegnia diclina and Saprolegnia parasitica on natural ecosystems and the aquaculture industry. Fungal Biol. Rev..

[3] Svoboda J., Mrugała A., Kozubíková-Balcarová E., Petrusek A. (2016). Hosts and transmission of the crayfish plague pathogen Aphanomyces astaci: A review. J. Fish. Dis..

[4] Edsman L.F.L., Gherardi F., Souty-Grosset C. (2010). *Astacus astacus*. The IUCN Red List of Threatened Species. https://www.iucnredlist.org/species/2191/9338388.

[5] Walker C.A., van West P. (2007). Zoospore development in the oomycetes. Fungal Biol. Rev..

[6] Gaulin E., Pel M.J.C., Camborde L., San-Clemente H., Courbier S., Dupouy M.-A., Lengellé J., Veyssiere M., Le Ru A., Grandjean F. (2018). Genomics analysis of Aphanomyces spp. identifies a new class of oomycete effector associated with host adaptation. BMC Biol..

[7] Nagasawa H. (2012). The crustacean cuticle: Structure, composition and mineralization. Front. Biosci. (Elite Ed.).

[8] Andersson M., Cerenius L. (2002). Analysis of chitinase expression in the crayfish plague fungus Aphanomyces astaci. Dis. Aquat. Org..

[9] Makkonen J., Jussila J., Kokko H. (2012). The diversity of the pathogenic Oomycete (Aphanomyces astaci) chitinase genes within the genotypes indicate adaptation to its hosts. Fungal Genet. Biol..

[10] Hochwimmer G., Tober R., Bibars-Reiter R., Licek E., Steinborn R. (2009). Identification of two GH18 chitinase family genes and their use as targets for detection of the crayfish-plague oomycete Aphanomyces astaci. BMC Microbiol..

[11] Vaaje-Kolstad G., Westereng B., Horn S.J., Liu Z., Zhai H., Sørlie M., Eijsink V.G.H. (2010). An Oxidative Enzyme Boosting the Enzymatic Conversion of Recalcitrant Polysaccharides. Science.

[12] Quinlan R.J., Sweeney M.D., Leggio L.L., Otten H., Poulsen J.-C.N., Johansen K., Krogh K.B.R.M., Jørgensen C.I., Tovborg M., Anthonsen A. (2011). Insights into the oxidative degradation of cellulose by a copper metalloenzyme that exploits biomass components. Proc. Natl. Acad. Sci. USA.

[13] Hemsworth G., Taylor E., Kim R., Gregory R.C., Lewis S.J., Turkenburg J., Parkin A., Davies G.J., Walton P.H. (2013). The Copper Active Site of CBM33 Polysaccharide Oxygenases. J. Am. Chem. Soc..

[14] Sabbadin F., Hemsworth G.R., Ciano L., Henrissat B., DuPree P., Tryfona T., Marques R.D.S., Sweeney S.T., Besser K., Elias L. (2018). An ancient family of lytic polysaccharide monooxygenases with roles in arthropod development and biomass digestion. Nat. Commun..

[15] Kracher D., Scheiblbrandner S., Felice A.K.G., Breslmayr E., Preims M., Ludwicka K., Haltrich D., Eijsink V.G.H., Ludwig R. (2016). Extracellular electron transfer systems fuel cellulose oxidative degradation. Science.

[16] Tan T.-C., Kracher D., Gandini R., Sygmund C., Kittl R., Haltrich D., Hallberg B.M., Ludwig R., Divne C. (2015). Structural basis for cellobiose dehydrogenase action during oxidative cellulose degradation. Nat. Commun..

[17] Bissaro B., Røhr Å.K., Müller G., Chylenski P., Skaugen M., Forsberg Z., Horn S.J., Vaaje-Kolstad G., Eijsink V.G.H. (2017). Oxidative cleavage of polysaccharides by monocopper enzymes depends on H_2_O_2_. Nat. Chem. Biol..

[18] Bissaro B., Streit B., Isaksen I., Eijsink V.G.H., Beckham G.T., DuBois J.L., Røhr Å.K. (2020). Molecular mechanism of the chi-tinolytic peroxygenase reaction. Proc. Natl. Acad. Sci. USA.

[19] Zhang H., Yohe T., Huang L., Entwistle S., Wu P., Yang Z., Busk P.K., Xu Y., Yin Y. (2018). dbCAN2: A meta server for automated carbohydrate-active enzyme annotation. Nucleic Acids Res..

[20] Notredame C., Higgins D., Heringa J. (2000). T-coffee: A novel method for fast and accurate multiple sequence alignment. J. Mol. Biol..

[21] Kumar S., Stecher G., Li M., Knyaz C., Tamura K. (2018). MEGA X: Molecular Evolutionary Genetics Analysis across Computing Platforms. Mol. Biol. Evol..

[22] Huson D.H., Richter D.C., Rausch C., Dezulian T., Franz M., Rupp R. (2007). Dendroscope: An interactive viewer for large phylogenetic trees. BMC Bioinform..

[23] Waterhouse A.M., Procter J., Martin D., Clamp M., Barton G.J. (2009). Jalview Version 2--a multiple sequence alignment editor and analysis workbench. Bioinformatics.

[24] Afgan E., Baker D., Batut B., van den Beek M., Bouvier D., Čech M., Chilton J., Clements D., Coraor N., Gruning B.A. (2018). The Galaxy platform for accessible, reproducible and collaborative biomedical analyses: 2018 update. Nucleic Acids Res..

[25] Patro R., Duggal G., Love M.I., Irizarry R.A., Kingsford C. (2017). Salmon provides fast and bias-aware quantification of transcript expression. Nat. Methods.

[26] Waterhouse A., Bertoni M., Bienert S., Studer G., Tauriello G., Gumienny R., Heer F.T., de Beer T.A.P., Rempfer C., Bordoli L. (2018). SWISS-MODEL: Homology modelling of protein structures and complexes. Nucleic Acids Res..

[27] McNicholas S., Potterton E., Wilson K.S., Noble M.E.M. (2011). Presenting your structures: The CCP4mg molecular-graphics software. Acta Crystallogr. D Struct. Biol..

[28] Sabbadin F., Urresti S., Henrissat B., Avrova A.O., Welsh L.R.J., Lindley P.J., Csukai M., Squires J.N., Walton P.H., Davies G.J. Secreted pectin monooxygenases drive plant infection by pathogenic oomycetes. Science.

[29] Filiatrault-Chastel C., Navarro D., Haon M., Grisel S., Herpoël-Gimbert I., Chevret D., Fanuel M., Henrissat B., Heiss-Blanquet S., Margeot A. (2019). AA16, a new lytic polysaccharide monooxygenase family identified in fungal secretomes. Biotechnol. Biofuels.

[30] Van Aalten D.M.F., Komander D., Synstad B., Gaseidnes S., Peter M.G., Eijsink V.G.H. (2001). Structural insights into the catalytic mechanism of a family 18 exo-chitinase. Proc. Natl. Acad. Sci. USA.

[31] Prakash N.A.U., Jayanthi M., Sabarinathan R., Kangueane P., Mathew L., Sekar K. (2010). Evolution, Homology Conservation, and Identification of Unique Sequence Signatures in GH19 Family Chitinases. J. Mol. Evol..

[32] Intra J., Pavesi G., Horner D.S. (2008). Phylogenetic analyses suggest multiple changes of substrate specificity within the Glycosyl hydrolase 20 family. BMC Evol. Biol..

[33] Gal-Mor O., Finlay B.B. (2006). Pathogenicity islands: A molecular toolbox for bacterial virulence. Cell. Microbiol..

[34] Gibbs G.M., Roelants K., O’Bryan M.K. (2008). The CAP superfamily: Cysteine-rich secretory proteins, antigen 5, and pathogene-sis-related 1 proteins--roles in reproduction, cancer, and immune defense. Endocr. Rev..

[35] Sidar A., Albuquerque E.D., Voshol G.P., Ram A.F.J., Vijgenboom E., Punt P.J. (2020). Carbohydrate Binding Modules: Diversity of Domain Architecture in Amylases and Cellulases From Filamentous Microorganisms. Front. Bioeng. Biotechnol..

[36] Frandsen K.E.H., Simmons T.J., DuPree T.J.S.P., Poulsen J.-C.N., Hemsworth G.R., Ciano L., Johnston E.M., Tovborg M., Johansen K., Von Freiesleben M.T.K.S.J.P. (2016). The molecular basis of polysaccharide cleavage by lytic polysaccharide monooxygenases. Nat. Chem. Biol..

[37] Forsberg Z., Sørlie M., Petrović D., Courtade G., Aachmann F.L., Vaaje-Kolstad G., Bissaro B., Røhr A.K., Eijsink V.G.H. (2019). Polysaccharide degradation by lytic polysaccharide monooxygenases. Curr. Opin. Struct. Biol..

[38] Chiu E., Hijnen M., Bunker R., Boudes M., Rajendran C., Aizel K., Olieric V., Schulze-Briese C., Mitsuhashi W., Young V. (2015). Structural basis for the enhancement of virulence by viral spindles and their in vivo crystallization. Proc. Natl. Acad. Sci. USA.

[39] Bouallegui Y. (2021). A Comprehensive Review on Crustaceans’ Immune System with a Focus on Freshwater Crayfish in Relation to Crayfish Plague Disease. Front. Immunol..

[40] De Lorenzo G., D’Ovidio R., Cervone F. (2001). The role of polygalacturonase-inhibiting proteins (PGIPS) in defense against pathogenic fungi. Ann. Rev. Phytopathol..

[41] Haeger W., Henning J., Heckel D.G., Pauchet Y., Kirsch R. (2020). Direct evidence for a new mode of plant defense against insects via a novel polygalacturonase-inhibiting protein expression strategy. J. Biol. Chem..

