# Supplementary material for: Lytic Polysaccharide Monooxygenases as Chitin-Specific Virulence Factors in Crayfish Plague
Source: Biomolecules. 2021 Aug 9;11(8):1180. doi: 10.3390/biom11081180 (PMC8393829; doi:10.3390/biom11081180)
Supplement: Supplementary file 1 [file biomolecules-11-01180-s001.zip › biomolecules-1318496-supplementary-CORRECT.pdf]

Supplementary Materials for:

Lytic polysaccharide monooxygenases as chitin-specific virulence factors in crayfish plague

Federico Sabbadin [1,*], Bernard Henrissat [2,3], Neil C. Bruce [1] and Simon J. McQueen-Mason [1,*]

1 Centre for Novel Agricultural Products, Department of Biology, University of York, York YO10 5DD, UK; neil.bruce@york.ac.uk
2 DTU Bioengineering, Technical University of Denmark, Søltofts Plads, 2800 Kongens Lyngby, Denmark;bernard.henrissat@gmail.com
3 Department of Biological Sciences, King Abdulaziz University, Jeddah 21589, Saudi Arabia
* Correspondence: federico.sabbadin@york.ac.uk (F.S.); simon.mcqueenmason@york.ac.uk (S.J.M.-M.)

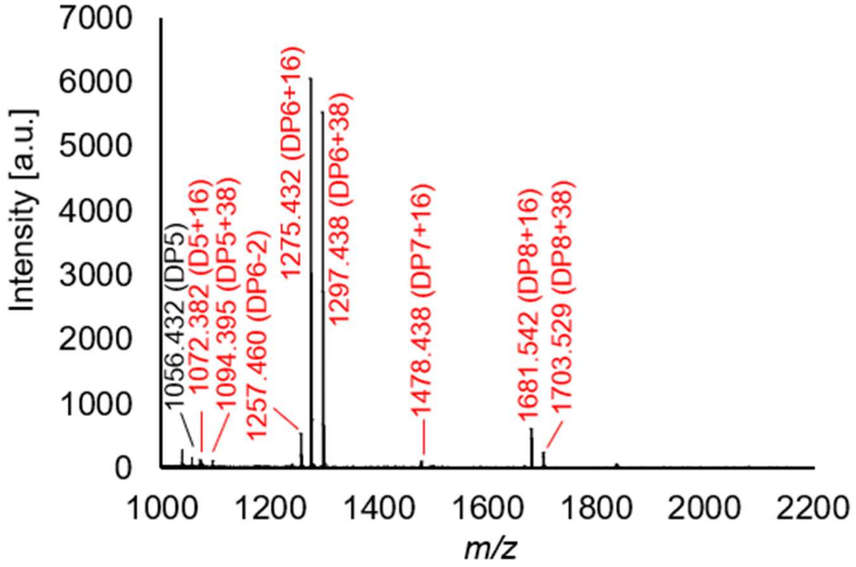

Figure S1. MALDI-TOF MS of activity assays of *Aa*AA15a with α-chitin.

MALDI-TOF MS analysis of in vitro activity assays with purified *Aa*AA15A, under the same experimental conditions as in Figure 5 e and f. The panel show the spectrum of products obtained after incubation of 4 mg mL^{-1} α-chitin with 2 μM *Aa*AA15A and 4 mM gallic acid. The main peaks correspond to mono- or di-sodiated adducts of C1-aldonic acids, imparting +16 or +38 m/z respectively, relative to the mono-sodiated unoxidized form. Native and oxidised species are marked in black and red, respectively.
